# Transmission Electron Microscopy Studies of Cellular Responses to Entry of Virions: One Kind of Natural Nanobiomaterial

**DOI:** 10.1155/2012/596589

**Published:** 2012-04-11

**Authors:** Zheng Liu, Shuyu Liu, Jinming Cui, Yurong Tan, Jian He, Jingqiang Zhang

**Affiliations:** ^1^Markey Center for Structural Biology, Purdue University, West Lafayette, IN 47907, USA; ^2^Guangzhou East Campus Lab Center, Sun Yat-sen University, Guangzhou 510006, China; ^3^Johns Hopkins School of Medicine, Baltimore, MD 21205, USA; ^4^University of Miami School of Medicine, Miami, FL 33136, USA; ^5^State Key Laboratory for Biocontrol, Sun Yat-sen University, Guangzhou 510275, China

## Abstract

Virions are one kind of nanoscale pathogen and are able to infect living cells of animals, plants, and bacteria. The infection is an intrinsic property of the virions, and the biological process provides a good model for studying how these nanoparticles enter into cells. During the infection, the viruses employ different strategies to which the cells have developed respective responses. For this paper, we chose *Bombyx mori* cypovirus 1 (BmCPV-1) interactions with midgut cells from silkworm, and severe acute respiratory syndrome (SARS) associated coronavirus interactions with Vero E6 cells, as examples to demonstrate the response of eukaryotic cells to two different types of virus from our previous studies. The bacteriophage-bacteria interactions are also introduced to elucidate how the bacteriophage conquers the barrier of cell walls in the prokaryotic cells to transport genome into the host.

## 1. Introduction

 A virus (including bacteriophages) is an infectious agent of small size and simple composition that can multiply only in living cells of animals, plants, or bacteria. The size of spherical virus usually ranges from ten nanometers to hundreds of nanometers in diameter. So they can be viewed as one kind of natural nanobiomaterial [1, 2]. A virus consists of single- or double-stranded nucleic acid and a protein shell, called a capsid. Some viruses also have an outer envelope composed of fatty materials (lipids) and proteins. The nucleic acid carries the virus's genome—its collection of genes—and may consist of either deoxyribonucleic acid (DNA) or ribonucleic acid (RNA). The protein capsid provides protection for the nucleic acid and may contain enzymes that enable the virus to enter its appropriate host cell. The host cells of viruses range from animals, plants, fungi, to bacteria and could be divided into two types: the eukaryotic cells and the prokaryotic cells.

Transmission electron microscopy (TEM) is a powerful tool to investigate microorganisms and has long been used in the discovery and description of viruses [3]. With appropriate sample preparation and application on a grid, the visual look of virions can be directly obtained. Beside traditional electron microscopy techniques such as negative staining, ultrathin sectioning, and immunoelectron microscopy [4], the relatively recently developed techniques such as cryoelectron microscopy (Cryo-EM) with single particle analysis that provides a new set of methods to investigate the 3D atomic resolution structures of macromolecules and cryo-electron tomography (CryoET) that allows the visualization of cellular structures under close-to-life conditions, are available to investigators [5–10]. Thus, TEM has been and continues to be valuable in elucidating mechanisms of virus attachment and replication in cells. Such information can be useful in the understanding of cellular response to viruses. In this paper, we discuss our previous studies on *Bombyx mori* cypovirus 1 (BmCPV-1) interactions with midgut cells from silkworm, and severe acute respiratory syndrome (SARS) associated coronavirus interactions with Vero E6 cells as examples to demonstrate the response of eukaryotic cells to two different types of viruses [11, 12]. The bacteriophage-bacteria interactions are also summarized to show how the bacteriophage conquers the barrier of cell walls in the prokaryotic cells to transport its genome into the host.

## 2. Entry of BmCPV into the Midgut Cells

**Figure 1:**
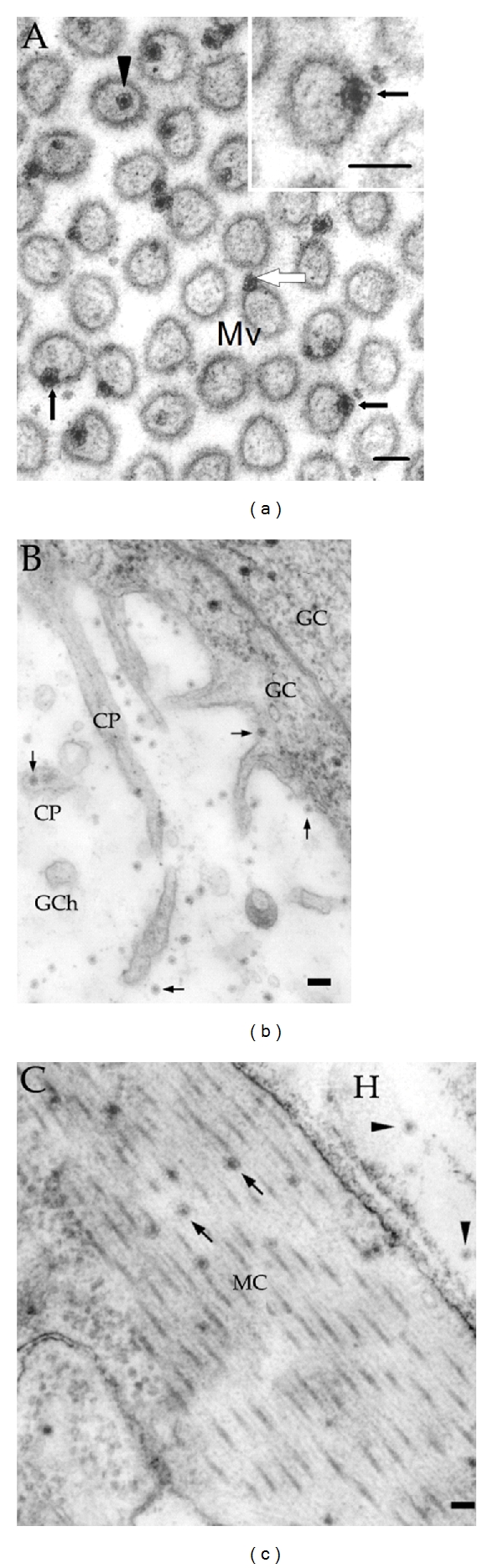
Electron micrographs of *Bombyx mori *midgut cells perorally inoculated with BmCPV-1. (a) Cross-section of microvilli (Mv) showing different stages of virus entry into midgut columnar cells. Intact virions were seen to adhere to (open arrow), penetrate (filled arrows), and be inside (arrowhead) the microvilli. Insert: higher magnification of a virion penetrating the membrane. (b) Virions (arrows) were detected in the goblet chamber (GCh) and in the cytoplasmic projections (CP) and the cytoplasm of the goblet cell (GC). (c) Virions (arrows) were distributed among myofibrils in the cytoplasm of midgut muscle cell (MC). Several virions (arrowheads) occurred in the haemocoel (H). Bars = 100 nm [11].

*Bombyx mori* cypovirus 1 (BmCPV-1), a member of the genus Cypovirus in the family Reoviridae, infects the silkworm (*Bombyx mori*) and is an important agricultural pathogen [13]. BmCPV particles have a diameter of about 70 nm and have long served as a model system to achieve high-resolution structures in cryoEM and single particle analysis because of their rigidity, high yield, and special surface features [14, 15]. The host cell of BmCPV is the midgut cells of silkworm. The midguts were collected for ultra-thin sectioning at different time points following the administration of virus-contaminated mulberry leaves. Electron microscopy observations showed the presence of virions both outside and inside the midgut cells at 3 hours postinoculation (Figure 1). It was obvious that the fibrous peritrophic membrane did not block the invasion of virions. Virions were seen adhering to the surface of microvilli, penetrating the plasma membrane, and settling themselves inside the microvilli (Figure 1(b)). The plasma membrane with embedded virions was discontinuous only at the virus penetration site and no obvious disruption of the membrane was observed. It was noted that all these virions retained their icosahedral integrity and seemed unaffected by the entry. Since no plasma membrane invaginations or cytoplasmic vesicles containing virions were detected, we excluded the possibility of virion internalization by means of viropexis. We summarized the process of entry of BmCPV-1 virions into columnar cells as follows. Initially, the virions recognized and attached themselves to the plasma membrane of microvilli by viral spikes, then the virions continued to interact with the membrane, resulting in a barely discernible membrane disruption at the site. Eventually, the virions penetrated through the microvilli and settled in the cytoplasm of columnar cells.

## 3. Entry of SARS Coronavirus into Vero E6 Cells by Membrane Fusion

**Figure 2:**
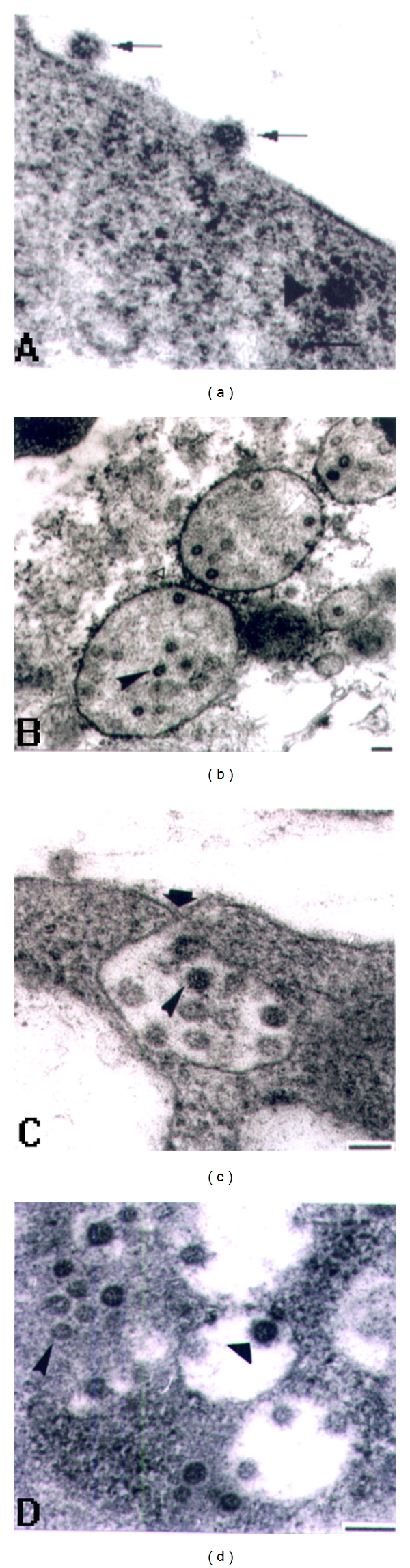
Severe acute respiratory syndrome-associated coronavirus (SARS CoV) in Vero E6 cell. (a) The virions attached themselves to the cell surface (arrow), and then their envelopes fused with cell membrane and the nucleocapsids entered the cell. The contours of these nucleocapsids were blurry after the virions lost their envelopes (arrowhead). (b) Nucleocapsids assembled in the swollen RER (arrow). Some ribosomes attached on the membrane of the RER. (c) Release of the virions from the cell. A smooth vesicle was fusing with the cell membrane (wide arrow); virions still located in the smooth vesicles (sharp arrow). (d) Budding of the nucleocapsids from the VMMV to the smooth vesicles. The SARS CoV nucleocapsids budded from the VMMV (sharp arrow) into the smooth vesicles and obtained spikes and envelopes (wide arrow). Bar = 100 nm [12].

SARS coronavirus, the pathagen causing the outbreak of severe acute respiratory syndrome (SARS) in 2003, belongs to the family Coronaviridae [16, 17]. Compared to BmCPV, SARS coronavirus possesses a lipid membrane with a diameter of about 100 nm while BmCPV has no membrane [18]. A sensitive host cell line of SARS coronavirus is Vero E6 cells that were derived from the kidney epithelial cells extracted from an African green monkey (*Chlorocebus sp.*) [12]. It could be seen with ultrathin sectioning and electron microscopy that the virions first attached themselves to the surface of host cell, then their envelopes fused with the cell membrane, and the whole nucleocapsids entered the cell. The contours of the nucleocapsids were blurred after the virions lost their envelopes (Figure 2(a)). After entry of the virus, the cell organelles began to work for the propagation of the virus. Ribosomes were recruited onto RER, capsid of the virus initially assembled in the chamber of ER, and then were enclosed in the virus morphogenesis matrix vesicle (VMMV) that came from RER (Figure 2(b)). Seven hours postinfection (p.i.), the formation of smooth vesicles at the Golgi apparatus was observed, then the nucleocapsids in VMMV were seen to bud into the smooth vesicles and acquire their envelopes in the sample of 24 hours p.i. (Figure 2(d)). Finally, the smooth vesicles fused with the cell membrane and virions were released (Figure 2(c)). Due to the size of about 100 nm in diameter and the obvious features on the envelopes, it is very easy to observe the virus under an electron microscope.

## 4. Bacteriophage-Host Interactions

**Figure 3:**
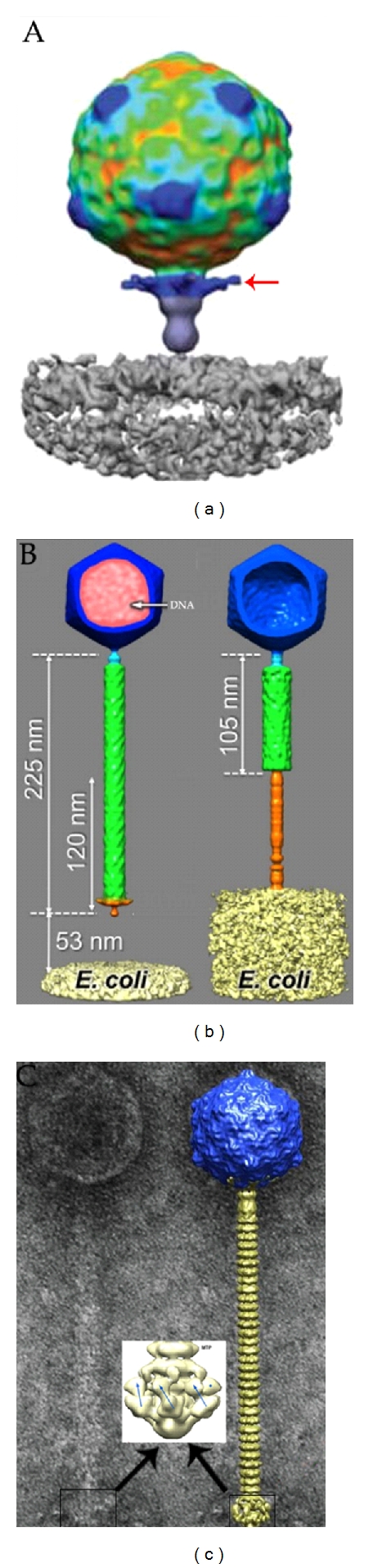
Observation of bacteriophage-host interaction with cryoelectron microscopy. (a) Cryo-electron tomography of podovirus P-SSP7 infecting *Prochlorococcus.* An infecting phage subtomograms with the portal vertex oriented to the cell surface. The phage tail fibers are extended horizontally (red arrow marked) [24]. (b) Conformational changes of phage P1 during tail contraction and DNA injection. The *E.coli* marked is only part of the cell, DNA marked pink stays in the capsid, and the tail is not contracted (left). Structure of the contracted phages has no DNA (right) [26]. (c) Electron micrograph of the virulent lactococcal phage p2. Overall structure of phage p2 (right) showing the capsid (blue, top), the tail, formed of rings of the major tail protein hexamers (gold), the globular baseplate (gold, bottom) (insert). The baseplate is reconstructed by single particles analysis. The receptor-binding protein (RBP) positions are identified by blue arrows or a blue dot. The RBP head is pointing upwards [29].

Unlike eukaryotic cells, bacteria, the host cell of bacteriophage, possess cell walls and do not contain a nucleus. It is broadly accepted that the phages release their genome into bacteria through the portal pore located at one vertex of the capsid and leave the empty capsid attached to surface of the host cell [19, 20]. During this process, one interesting step is how the phage breaches the hydrophobic barrier of the membrane(s) and the polymeric sugar structure of the cell wall. Recently this issue has benefited from the developments in cryo-electron microscopy (cryo-EM) and cryo-electron tomography (cryo-ET) approaches which now offer a comprehensive and dynamic view of the in situ crosstalk between the phage and its host even at nanometer scale [21, 22]. The structures of the Podoviridae phage epsilon 15 with its host *Salmonella anatum* [23] and the marine podovirus with its host *Prochlorococcus* [24] suggest that phage binding to its outer membrane receptor triggers the opening of the tail hub (Figure 3(a)). The inner core then exits from the capsid and forms a tube across the periplasm through which the DNA is transported. Coliphage T4 [25] and P1 (Myoviridae) [26] recognize first their host saccharidic receptors via their long tail fibers, which induces a conformational change of the baseplate that exposes the short tail fibers and ensures a definitive docking of the phage onto the host cell wall (Figure 3(b)). In parallel, the tail contracts and the dsDNA is ejected. For noncontractile-tailed siphophages, such as *E. coli* T5 [27], *Bacillus subtilis* SPP1 [28], and lactococcal phage p2 [29], saccharidic chains are recognized nonspecifically by phage components in an initial reversible step, followed by the irreversible specific docking of the tail fiber protein onto the host receptor, the FhuA porin, or the extramembrane domain of YueB, respectively (Figure 3(c)).

## 5. Conclusions

Electron microscopy is a powerful tool to visualize the ultrastructure of cell organelles and virus particles and to study virus-cell interactions. The conventional ultra-thin sectioning technology could capture different steps of virus infection of the host cell. Recently, the cryo-thin section technology has overcome the loss of integrity of cells caused by fixation during sample preparation for normal thin section. CryoEM techniques, including the freezing of the sample at liquid nitrogen temperature, imaging at low dose (~20 e/Å^2^), and improved image processing algorithms, also provide promising approaches to study the virus particles at atomic resolution and the virus-cell interaction *in vivo*.

Virus-cell interaction, an essential step for virus infection, is a fast process. Viruses employ different strategies based on their own architecture and the type of the host cell. The prokaryotic cells have a cell wall which is strong and relatively thick. Bacterial viruses (bacteriophages) have therefore evolved tails to bind to the cell and then to eject the genome into the host cell. Eukaryotic cells do not have cell walls; they have only lipid bilayer cell membranes with associated proteins. Viruses take advantage of these structures in a number of ways. The simplest is membrane fusion, where the virion membrane fuses with the cell membrane directly or through mediation by a specific receptor, and the virion nucleoprotein complex is delivered into the cell cytoplasm directly. All enveloped viruses appear to share the fusion mode of entry. Most nonenveloped viruses, such as the dsDNA adenoviruses (Adenoviridae) and the simpler dsRNA *Bombyx mori* cypovirus 1, enter cells via penetration and endocytosis vesicles.
